# Adiponectin and Thyroid Cancer: Insight into the Association between Adiponectin and Obesity

**DOI:** 10.14336/AD.2020.0919

**Published:** 2021-04-01

**Authors:** Yuanyuan Zhou, Ying Yang, Taicheng Zhou, Bai Li, Zhanjian Wang

**Affiliations:** ^1^Department of Endocrinology and Metabolism, The Second People’s Hospital of Yunnan Province, Fourth Affiliated Hospital of Kunming Medical University, Kunming, China.; ^2^Department of Endocrinology and Metabolism, Sixth Affiliated Hospital of Kunming Medical University, The People’s Hospital of Yuxi City, Yuxi, China.; ^3^School of Medicine, Yunnan University, Kunming, China.; ^4^Department of Endocrinology and Metabolism, The Third Hospital of Hebei Medical University, Shijiazhuang, China.

**Keywords:** thyroid cancer, adiponectin, obesity

## Abstract

In recent decades, the incidence and diagnosis of thyroid cancer have risen dramatically, and thyroid cancer has now become the most common endocrine cancer in the world. The onset of thyroid cancer is insidious, and its progression is slow and difficult to detect. Therefore, early prevention and treatment have important strategic significance. Moreover, an in-depth exploration of the pathogenesis of thyroid cancer is key to early prevention and treatment. Substantial evidence supports obesity as an independent risk factor for thyroid cancer. Adipose tissue dysfunction in the obese state is accompanied by dysregulation of a variety of adipocytokines. Adiponectin (APN) is one of the most pivotal adipocytokines, and its connection with obesity and obesity-related disease has gradually become a hot topic in research. Recently, the association between APN and thyroid cancer has received increasing attention. The purpose of this review is to systematically review previous studies, give prominence to APN, focus on the relationship between APN, obesity and thyroid cancer, and uncover the underlying pathogenic mechanisms.

In recent decades, the incidence and diagnosis of thyroid cancer have risen sharply. According to data from the International Agency for Research on Cancer (IARC), as of 2018, the global incidence of thyroid cancer was approximately 6.7/10 million [1]. Thus, thyroid cancer has become the most common endocrine cancer in the world [2-5].

Based on histological features, thyroid cancers can be categorized into well-differentiated thyroid carcinomas (WDTCs), which account for approximately 95% of all thyroid cancers and include papillary thyroid carcinoma (PTC) and follicular thyroid carcinoma (FTC); anaplastic or thyroid carcinoma (ATC), which is relatively rare but highly invasive and usually involves a poor prognosis; and medullary thyroid carcinoma (MTC), which originates from parafollicular cells (C cells), which can secrete calcitonin and serotonin, and can be sporadic or familial [6-8].

Most thyroid cancers develop slowly and are difficult to detect. Although the popularization of ultrasound technology in recent years has greatly improved the diagnosis rate of thyroid nodules and thyroid cancer [9], identifying the specific pathogenesis of thyroid cancer is the foundation for early prevention and treatment.

Although numerous studies have been conducted, the etiology of thyroid cancer has still not been fully delineated. Currently, factors such as female sex [10], radiation [11, 12], dietary iodine content [13], genetic or hereditary conditions [8, 14-16], and a history of benign thyroid disease [17, 18] are considered risk factors for thyroid cancer. Interestingly, the chronic epidemic of overweight and obesity has accompanied the dramatic increase in the incidence rate of thyroid cancer [19-21]. Existing epidemiological studies and meta-analyses have shown that overweight and/or obesity caused by changes in the environment and lifestyle may be implicated in the pathogenesis of thyroid cancer [22-25]. More importantly, in the obese state, dysfunction of adipose tissue, a potential "endocrine organ", is accompanied by dysregulation of a variety of cytokines (including adipocytokines) [26, 27]. Adiponectin (APN) is one of the most pivotal adipocytokines, and its connection with obesity and thyroid disease has gradually become a hot topic in research.

In this review, we focus on APN as a core factor, pay attention to the relationship between APN and thyroid cancer and explore the effects of APN and obesity on thyroid cancer and the possible mechanisms involved.

## Brief introduction to APN

### Structure of APN

Under normal physiological conditions, adipose tissue not only stores energy but also is an active endocrine organ that can secrete a variety of active adipokines, such as APN, leptin, and resistin. However, in the obese state, adipose tissue dysfunction, which is accompanied by an imbalance in the secretion of adipokines and causes obesity-related diseases, occurs.

APN, one of the most widely studied adipokines, is mainly secreted by adipocytes [26, 28, 29]. Other cell types, such as cardiomyocytes, skeletal muscle cells, and lymphocytes, can also produce APN [30-33]. Monomeric APN is 244 amino acids long and is encoded by the ADIPOQ gene on human chromosome 3q27. It consists of a signal peptide region at the NH2 terminus, a variable region, a collagen domain, and a globular domain at the COOH terminus [34].

APN is the most abundant protein in human plasma, accounting for 0.05% of total human plasma protein, and its concentration is approximately 3 ~ 30 g/mL [29]. Full-length APN circulates as three main isoforms: low molecular weight (LMW)-APN, which results from the trimerization of monomeric APN, middle molecular weight (MMW)-APN, which is formed by through two trimers, and high molecular weight (HMW)-APN, which is formed by multiple trimers [35]. Each APN isoform exerts distinct biological functions in different tissues by activating different signal transduction pathways [36]. Among them, HMW-APN is believed to be the most biologically active isoform [37].

Moreover, globular APN, which is generated from the proteolytic cleavage of the COOH terminus, is also present in human plasma at a low concentration [38].

### APN Receptors

AdipoR1 and AdipoR2, classical receptors for APN, consist of 7 transmembrane domains and have distinct binding affinities for different APN isoforms [39].

AdipoR1 is almost ubiquitous; it is abundantly expressed in skeletal muscle and endothelial cells and has a high affinity for globular APN and a low affinity for full-length APN. However, AdipoR2 is expressed mainly in the liver and binds full-length APN and globular APN with moderate affinity [39-41]. In addition, a nonclassical APN receptor, T-cadherin, which lacks a transmembrane domain, mainly binds to HMW-APN and is involved in cell-cell interactions and signaling in a calcium-dependent manner [39-41], has also been identified. APN binds to corresponding receptors and activates downstream signaling pathways to perform a wide range of biological functions. Studies have demonstrated that APN can exert anti-inflammatory [42], antidiabetic [43], anti-insulin resistance [44], myocardial protective [45], anti-atherosclerosis [46], and immunoregulatory effects [47, 48].

The signaling pathways related to APN will be described in the following chapters.

## Association between APN and obesity

### The Central Role of Chronic Low-Grade Inflammation in Obesity

Undoubtedly, inflammation is the major typical feature of obesity. Obesity-related inflammation seems to be triggered, although other metabolic factors may be involved in the course [49]. In obesity, changes in the surrounding environment and modifications of the paracrine function of adipocytes result from changes in fat pad size and adipocytes. Hypertrophic adipocytes secrete tumor necrosis factor-α (TNF-α), which stimulates preadipocytes to produce monocyte chemotactic protein-1 (MCP-1). MCP-1 and other cytokines, such as TNF-α, interleukin-1 (IL-1), and interleukin-6 (IL-6), can contribute to the recruitment of macrophages to adipose tissue [50, 51].

In addition, either increased production of leptin or reduced secretion of APN from adipocytes may promote accumulation of macrophages in adipose tissue and enhance adhesion of macrophages to endothelial cells [52]. Regardless of the stimulus, once macrophages are activated, they, along with adipocytes and other cells, further produce more inflammatory cytokines, aggravate dysfunction of adipocytes, and recruit more macrophages to adipose tissue. Consequently, a vicious feedback loop is initiated and perpetuated [50, 53, 54].

More importantly, as research has progressed, innate immune cells besides macrophages (such as monocytes and natural killer cells) and adaptive immune cells (such as T and B lymphocytes) have also been identified to participate in adipose tissue inflammation [55, 56]. Hence, the central role of macrophages in the inflammatory response in adipose tissue is well established [57].

### Anti-Inflammatory Properties of APN

**Figure 1.**
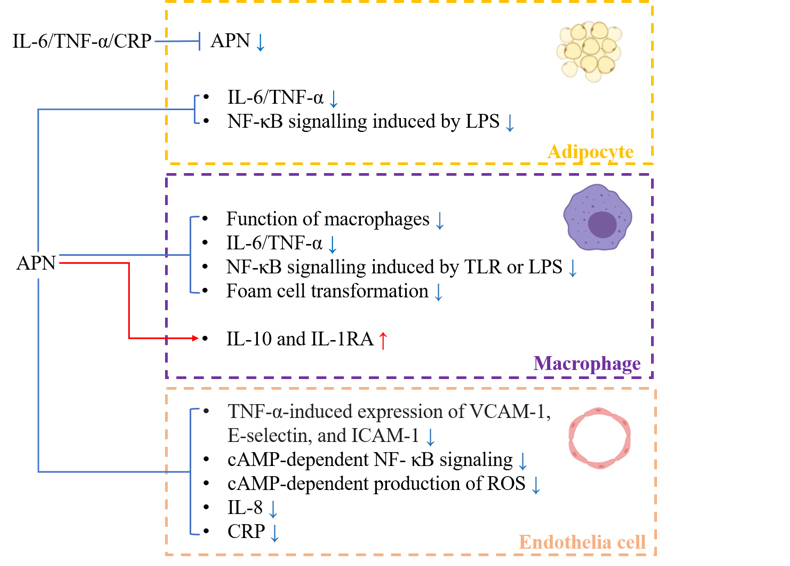
The anti-inflammatory properties of APN. APN exerts its anti-inflammatory properties in different cell types through mechanisms as follows. In adipocytes, a bi-directional regulation has been shown to exist between APN and pro-inflammatory factors, such as TNF-α, IL-6, and CRP. APN treated macrophages exhibited impaired function of phagocytic activity, suppressed foam cell transformation, negative regulation of NF-κB signaling, decreased expression of TNF-α or IL-6, and increased release of IL-10 and IL-1RA. In endothelial cells, APN can negatively regulate the expression of VCAM-1, E-selectin, ICAM-1, CRP, and IL-8, and suppress endothelial NF-κB activation and ROS production. The red arrow indicates promotion, and the blue arrow indicates inhibition. Abbreviations: APN adiponectin, TNF-α tumor necrosis factor-α, IL-6 interleukin-6, CRP C-reactive protein, NF-κB nuclear factor kappa-B, IL-10 interleukin-10, IL-1RA interleukin-1 receptor agonist, VCAM-1 vascular cell adhesion molecule 1, ICAM-1 intracellular adhesion molecule 1, IL-8 interleukin-8, ROS reactive oxygen species.

Classical proinflammatory cytokines, such as C-reactive protein (CRP), TNF-α, and IL-6, are biomarkers of obesity-related chronic inflammation [42, 58]. Several clinical studies have supported the inverse relationship between APN and these inflammatory factors, especially in obese individuals [59-62]. Moreover, accumulative evidence from *vitro* and *vivo* studies has suggested that APN acts as an anti-inflammatory factor in different cell types, such as adipocytes, inflammatory cells, and endothelial cells, through several mechanisms that have been summarized in Figure 1.

In adipocytes, APN will downregulate the activation of nuclear factor kappa-B (NF-κB) signaling that is induced by lipopolysaccharides (LPS), as well as the expression of IL-6 [63]. In addition, APN knock-out mice exhibited high levels of TNF-α mRNA in adipose tissue [64]. In turn, CRP [65], TNF-α [66], and IL-6 [67] have been confirmed to suppress APN production in adipocytes.

Moreover, the negative correlation of APN and inflammatory cells is mainly generated from data from studies conducted in macrophages, given the central role of macrophages in the inflammatory response in adipose tissue [57, 68]. On one hand, APN not only suppresses the function of macrophages, including phagocytic activity [69], proliferation, plasticity, and polarization [68], but also lowers the abundance of acyl-CoA: cholesterol acyltransferase-1, reduces the expression of class A macrophage scavenger receptor, interferes with the absorption and storage of lipids, and ultimately leads to the suppressed formation of foam cells [70]. On the other hand, APN negatively regulates NF-κB signaling that is induced by LPS [63] or the Toll-like receptor family (TLR) [71], inhibits the production of TNF-α and IL-6 [63], and promotes the synthesis of the anti-inflammatory mediators interleukin-10 (IL-10) and interleukin-1 receptor agonist (IL-1RA) [72].

In addition, a growing amount of evidence has shown a close link between APN and endothelial dysfunction. APN can inhibit TNF-α-induced expression of vascular cell adhesion molecule 1 (VCAM-1), E-selectin, and intracellular adhesion molecule 1 (ICAM-1) [73] and can decrease the synthesis of CRP [74] and interleukin-8 (IL-8) [75] in endothelial cells. Furthermore, APN can suppress endothelial NF-κB signaling and the production of reactive oxygen species (ROS) through a cAMP-dependent pathway [76, 77], suggesting that APN has an endothelial protective effect.

### Evidence from Related Research on APN and Obesity-Related Diseases

As mentioned previously, adipocytes are the main source of APN. A great number of studies have shown that the levels of circulating APN in obese people are significantly decreased compared to those in control subjects [78] and that weight loss leads to increased APN levels [79]. In addition, data from numerous studies have confirmed that APN is associated with other obesity-related diseases, such as type 2 diabetes [43, 80], insulin resistance [44, 81], and cardiovascular diseases, such as coronary heart disease [45, 82], atherosclerosis [46], angina pectoris [83], and hypertension [84]. Correspondingly, the expression of AdipoR1 in adipose tissue is decreased in obese individuals compared to control subjects, whereas expression of AdipoR1 increases after weight loss, suggesting that the sensitivity of obese individuals to APN is decreased [85].

Apart from these obesity-related diseases described above, obesity has been widely studied in the context of malignancies. Increasing evidence suggests that overweight and obesity are risk factors for multiple malignancies. Data from the International Agency for Research on Cancer (IARC) suggest that obesity and overweight are linked to at least 13 cancers [86], especially tumors closely related to endocrine hormones, such as breast cancer [87, 88], endometrial cancer [89-93], ovarian cancer [94, 95] and prostate cancer [96, 97]. Here, we aim to focus on APN and shed light on the association between APN and obesity and their role in the occurrence, development, and progression of thyroid cancer.

## Obesity and thyroid cancer

### Research Progress on Obesity and Thyroid Cancer

Growing evidence has indicated that being overweight or obese increases the risk of thyroid cancer [98-103]. For example, a meta-analysis conducted by Schmid D et al. reported that, compared with lean individuals, 25% and 55% greater risks of thyroid cancer were found in individuals with overweight and in individuals with obesity, respectively [98]. In the United States, one in six PTC in 60-year-old adults is attributable to overweight or obesity [99]. Being overweight or obese not only increases the risk of thyroid cancer but also seems to be related to specific thyroid cancer pathological types [22, 104]. A higher body mass index (BMI) can increase the risk of differentiated thyroid carcinomas (DTC) (including FTC and PTC) [100]. A large case-control study from China also identified BMI, body surface area (BSA), and body fat percentage (BF%) as positive risk factors for DTC and suggested that these factors might be affected by age and gender. However, these factors were not significant risk factors for MTC [101]. Additionally, a higher BMI is not only significantly associated with larger tumor size and a greater risk of multifocal tumor [102] but also increases the incidence of postoperative complications, such as postoperative hemorrhage and accessory nerve injury [103].

Undeniably, the incidence rate of thyroid cancer is higher in women than in men. Furthermore, some studies have suggested that gender is a vital factor that effects the relationship between obesity and thyroid cancer [105-108]. Several studies have shown that obesity increases the risk of thyroid cancer regardless of gender or only in men [109], whereas the majority of population-based studies indicate that obesity increases the risk of thyroid cancer in women [110-112].

**Figure 2.**
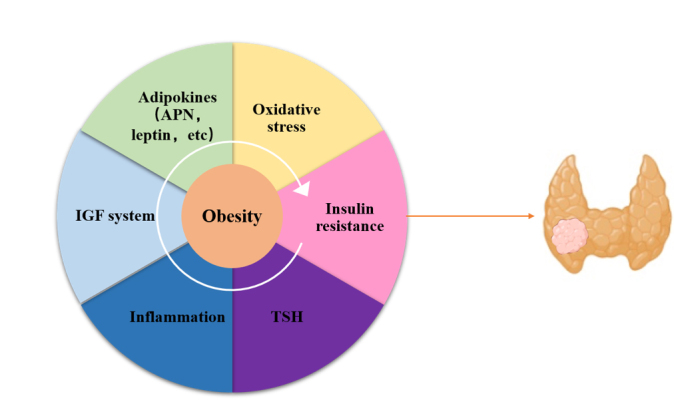
The possible mechanisms underlying obesity and thyroid cancer. Elevated serum TSH and other obesity-related comorbidities, including abnormal insulin resistance, chronic low degree inflammation, oxidative stress, abnormalities of the IGF system, and altered secretion of adipokines, could be the possible carcinogenic mechanism underlying obesity and thyroid cancer. Moreover, all of these factors may interplay with each other, forming a vicious cycle. Abbreviations: TSH thyroid-stimulating hormone, IGF insulin-like growth factor.

Additionally, BMI seems to be significantly affected by age. A study in Pakistan showed that although patients with thyroid cancer were mostly obese women, obese patients > 45 years of age accounted for 72.8% of all patients [110]. A recent study conducted by He et al. [101] suggested that an increased BMI value is accompanied by a risk for DTC in women of all ages and men under 50 years old, but not in men over the age of 50. Interestingly, some studies have shown that overweight or obesity status in early adulthood is also closely related to the risk of thyroid cancer, particularly in female patients [113, 114].

Therefore, in addition to obesity itself, factors such as gender, age, and the period of obesity in adulthood should be considered essential comprehensive factors that affect the relationship between obesity and thyroid cancer.

### The Possible Mechanisms underlying Obesity and Thyroid Cancer

Although the complex molecular relationship underlying obesity and thyroid cancers has not been fully elucidated, some pathogenic mechanisms have been proposed. Thyroid-stimulating hormone (TSH) can physiologically regulate cell growth and function by binding to its receptor [115]. Previously, a number of studies based on population have confirmed that BMI is positively correlated with serum TSH and free triiodothyroxine (FT3), even among euthyroid individuals [116-118]. However, several studies have associated the elevated TSH with insulin resistance, but not BMI [119, 120]. Notably, the majority of clinical data showed a positive correlation link between serum TSH and thyroid carcinogenesis [121-123], and TSH suppression is strongly recommended for high-risk patients and patients with DTC after surgery according to the American Thyroid Association (ATA) [124]. Nevertheless, the existing data cannot strongly support TSH hormone as an independently pathogenic factor in the association between obesity and thyroid cancer [23, 112].

Apart from TSH, thyroid cancers share some general mediator factors with other obesity-related cancers, which has been discussed in many excellent reviews [125-127]. Chronic low-grade inflammation, in addition to being a characteristic of adipose tissue in the obese state, also exists in the tumor microenvironment. Furthermore, obesity is often associated with metabolic defects and is accompanied by abnormal insulin resistance, oxidative stress, abnormalities of the insulin-like growth factor (IGF) system and altered secretion of adipokines. All of these factors may not act independently, but in combination to promote the development of malignant tumors (shown in Fig. 2).

**Table 1:** The summary of studies on APN and thyroid cancer.

Title	First author	Year	Country	Journal	Results
*Serum adiponectin and progranulin level in patients with benign thyroid nodule or papillary thyroid cancer ^[132]^*	Kwon H	2020	Korea	Endocrinol Metab (Seoul)	Serum APN levels showed no significant difference between benign and PTC groups
*Circulating adipokines and metabolic setting in differentiated thyroid cancer ^[129]^*	Mele C	2019	Italy	Endocr Connect	APN levels were lower in DTC compared to BTD group and controls. In parallel, HOMA-IR was higher in DTC than BTD and control group
*Concentrations of selected adipokines, interleukin-6, and vitamin d in patients with papillary thyroid carcinoma in respect to thyroid cancer stage^[130]^*	Warakomski J	2018	Poland	Int J Endocrinol	No significant relationships between serum concentrations of APN and tumor size in PTC was found. However, significantly decreased APN concentrations were observed in PTC patients with metabolic syndrome compare to patients without metabolic syndrome
*Adipokines and inflammation markers and risk of differentiated thyroid carcinoma: The EPIC study ^[131]^*	Dossus L	2018	France	Int J Cancer	A possible negative association of TC risk with prediagnostic circulating levels of APN in women
*Lack of association between serum adiponectin/leptin levels and medullary thyroid cancer^[133]^*	Abooshahab R	2016	Iran	Asian Pac J Cancer Prev	levels of APN in patients with MTC did not remarkably change when compared with the normal control group
*Serum adiponectin and insulin-like growth factor 1 in predominantly female patients with thyroid cancer: association with the histologic characteristics of the tumor ^[134]^*	Pazaitou-Panayiotou K	2016	Greece	Endocr Pract	IGF-1, IGFBP-3, and APN levels were similar among different histologic types of thyroid carcinoma. However, IGF-1 to APN and IGF-1 to (APN×IGFBP-3) ratios, they were independently associated with tumor size
*Expression and biologic significance of adiponectin receptors in papillary thyroid carcinoma ^[142]^*	Cheng SP	2013	Taiwan, China	Cell Biochem Biophys	Overexpression of AdipoRs was observed in some tumor tissues of PTC and was associated with a better prognosis
*Circulating adiponectin is inversely associated with risk of thyroid cancer: in vivo and in vitro studies ^[128]^*	Mitsiades N	2011	United States	J Clin Endocrinol Metab	A significant inverse association between circulating APN levels and risk of thyroid carcinoma, and particularly PTC. Although human thyroid tumor tissues express AdipoRs, recombinant APN did not have any substantial effect on cell proliferation or survival in thyroid cancer cell lines *in vitro*

Abbreviation: APN adiponectin, PTC papillary thyroid carcinoma, DTC differentiated thyroid carcinoma, BTD benign thyroid disease, HOMA-IR homeostasis model assessment of insulin resistance, TC thyroid cancer, MTC medullary thyroid carcinoma, IGF-1 insulin-like growth factor 1, IGFBP-3 IGF-binding protein 3, AdipoRs adiponectin receptors.

## APN and thyroid cancer

As mentioned above, obesity increases the risk of thyroid cancer and contributes to thyroid carcinogenesis through several mechanisms, including the abnormal secretion of APN. Recently, some progress regarding APN in the field of thyroid cancer has been achieved. Here, this review will explore the relationship between APN and thyroid cancer in-depth by reviewing previous related literature. A summary of studies on APN and thyroid cancer is shown in Table 1.

### Research Progress on APN in the Peripheral Circulation and Thyroid Cancer

In 2011, Mitsiades et al. [128] first explored the relationship between APN and thyroid cancer and found that circulating APN levels in patients with thyroid cancer (mostly PTC) were lower than those in healthy controls. Similarly, a study conducted by Mele et al. [129] illustrated that patients with DTC (including PTC and FTC) have lower APN levels than those with benign thyroid disease and healthy controls.

**Figure 3.**
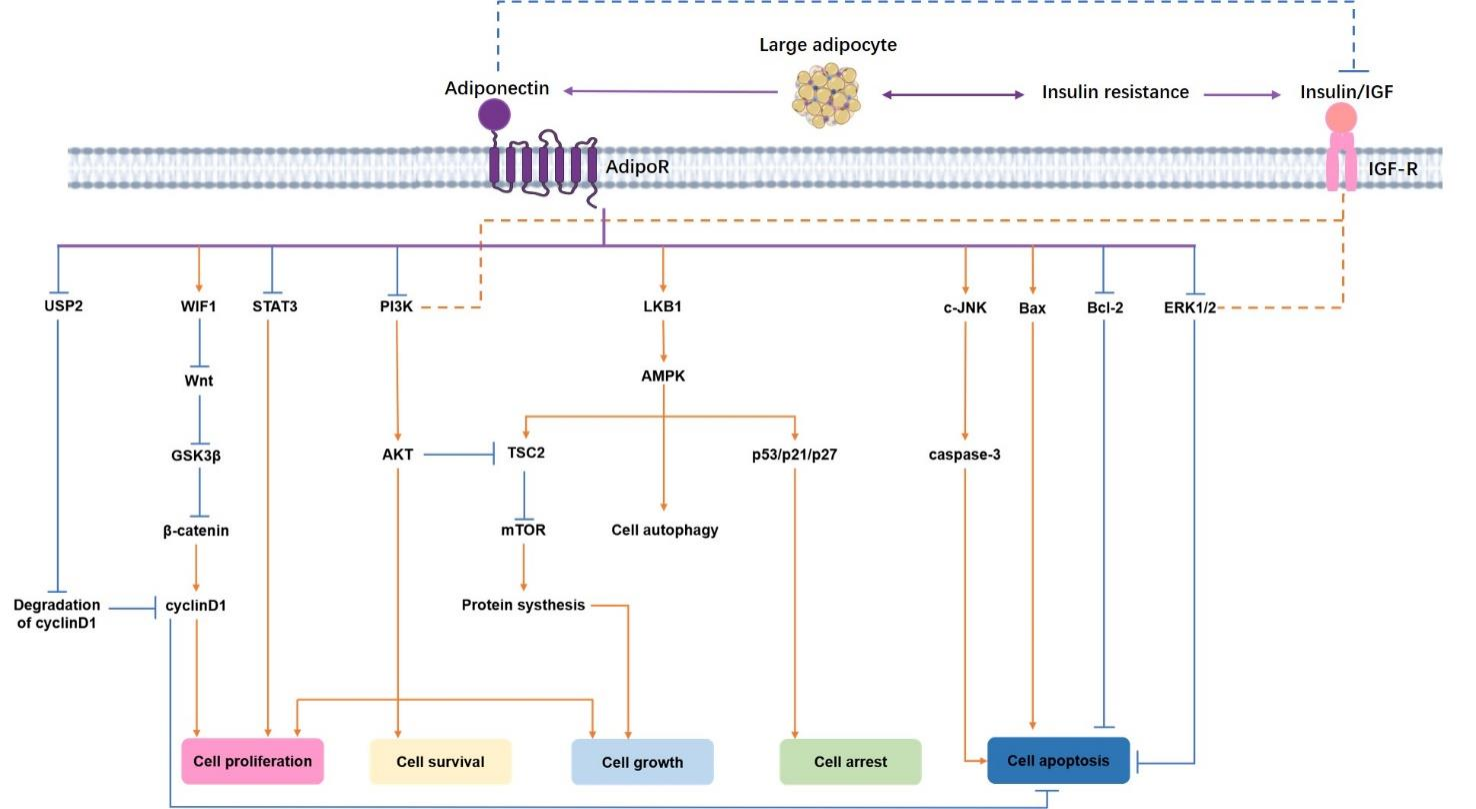
The directly and indirectly anti-cancer pathways of APN. Binding to AdipoR1/2, APN could initiate a series of directly anti-cancer pathways, including inhibition of PI3K/AKT, ERK1/2, Wnt/GSK3β/β-catenin, STAT3, USP2, and Bcl-2 signals; as well as activation of AMPK by LKB1, then up-regulates TSC2, p53/p21/p27 and promotes cell autophagy. Moreover, the tumorigenicity of the IGF axis has been confirmed to be partially through ERK and PI3K/AKT pathways, which are belonging to the downstream signaling of the APN. Since APN can increase insulin sensitivity, it is reasonable to believe that the complexity of the APN-related signaling network has been added by the possible indirect association between APN, insulin resistance, and the abnormal IGF system. The blue solid arrows and red solid arrows indicate inhibitory and stimulatory effects in the direct pathway, respectively. In turn, the blue dotted lines and red dotted lines indicate the inhibitory and stimulatory effects in the indirect pathway, respectively. Abbreviations: APN adiponectin, PI3K/AKT phosphatidylinositol 3-kinase/protein kinase B, ERK1/2 extracellular signal-regulated kinases 1 and 2 Wnt wingless-type protein, GSK3β glycogen synthase kinase 3β, STAT3 signal transducer and activator of transcription 3, USP2 ubiquitin-specific protease 2, AMPK 5’ adenosine monophosphate-activated protein kinase, LKB1 liver kinase B1, TSC2 tuberous sclerosis complex 2, IGF insulin-like growth factor.

Considering the close connection between APN, metabolic syndrome and obesity, Warakomski et al. [130] tried to investigate the association between metabolic syndrome, APN and thyroid cancer. The results from this study showed that significantly decreased APN concentrations are observed in PTC patients with metabolic syndrome compared to patients without metabolic syndrome, whereas the level of circulating APN has no effect on tumor size or progression. In 2018, the European Prospective Investigation into Cancer and Nutrition (EPIC) initiated a large-scale prospective case-control study that eventually included a total of 475 patients with thyroid cancer (including 363 patients with PTC, 84 patients with FTC and 28 patients with thyroid cancer not otherwise specified) and 1,016 matched healthy controls to assess the relationship between the prediagnostic level of APN and the risk of thyroid cancer. The results suggested that thyroid cancer risk in women may be negatively associated with the prediagnostic circulating level of APN [131]. However, a recent study showed that the serum APN in PTC had no significant difference compared with benign groups [132].

Notably, the results from Abooshahab et al. [133] showed that the levels of APN in patients with MTC were not markedly changed compared with those in normal controls. Pazaitou-Panayiotou et al. [134] evaluated circulating APN levels in different types of thyroid cancer, including PTC, FTC, and MTC, but no significant differences were detected.

According to the results from the abovementioned research, the correlation between the level of APN in the peripheral circulation and thyroid cancer seems to be complex. Likewise, this phenomenon also occurs in other endocrine cancers. Although data from the majority of previous studies have supported that circulating APN levels are negatively related to the risk, initiation, and progression of these endocrine cancers, some controversial findings still exist [135]. For example, data from a nested case-control study showed that concentrations of APN had no significant association with prostate cancer risk and aggressiveness [136]. Another research based on the Japanese population suggested a positive relationship between levels of APN and prostate specific antigen (PSA) and a higher risk of prostate cancer in overweight men with higher levels of APN [137]. Furthermore, Housa et al. [138] found that compared with organ-confined prostate cancer, levels of circulating APN are higher in locally advanced cancer. More interestingly, circulating APN has been demonstrated to be positively correlated with the all-cause mortality rate in most clinical diseases, including breast cancer and colorectal cancer [139-141].

Therefore, the effects of circulating APN on thyroid cancers may be varied according to the types of cancer, even the same type of cancer at different stages. In addition, study methods, characteristics of the test subjects, such as gender, age, obesity or not, and other unknown factors, will have an impact on the research results to a certain extent.

### Effect of APN on Thyroid Tumor Cell Lines

In addition to clinical studies, some *in vitro* studies have been conducted to explore the effects of APN intervention on thyroid tumor cell lines.

Mitsiades et al. [128] showed that both low and high doses of APN have no effect on the proliferation, cell cycle or apoptosis of BHP7 or SW579 cells or on the expression of p53 or p21.

Another study indicated that APN can activate 5’ adenosine monophosphate-activated protein kinase (AMPK) phosphorylation in the K1 and B-CPAP cell lines, but the effect of APN on cell function has not been further identified [142]. Several previous studies on other malignancies have shown that AMPK activation can inhibit cell growth and proliferation. Therefore, carrying out further follow-up is necessary.

### Studies on AdipoRs in Thyroid Cancer

Considering that APN mainly exerts its biological effect through AdipoRs and that altered expression of AdipoRs has also been confirmed to be related to some malignant tumors, it is necessary to investigate the relationship between AdipoRs and thyroid cancer.

RT-qPCR results from the study conducted by Mitsiades et al. [128] illustrated that both AdipoR1 and 2 are detected in thyroid cancer cell lines (SW579 and BHP7), thyroid cancer tissues (including 32 PTC samples, 6 FTC samples, 1 ATC sample and 1 MTC sample) and 4 normal thyroid tissues. Moreover, the expression levels of AdipoR1 and AdipoR2 in PTC tissues are lower than those in normal thyroid tissues. However, another study found that the protein levels of AdipoR1 and AdipoR2 are increased in some PTC samples compared with adjacent normal thyroid tissues. Further immunohistochemical staining results showed that AdipoR1 and AdipoR2 expression is barely detectable in tumor-adjacent normal thyroid tissue; however, AdipoR1 expression is found in 27% of thyroid cancer tissues, and AdipoR2 expression is found in 47% of such tissues. Interestingly, negative expression of two AdipoRs is significantly associated with extrathyroid invasion, polycentricity, and higher TNM stages. The disease-free survival rate of patients with negative AdipoR1 and AdipoR2 expression is decreased compared with that of patients with positive expression [142].

Thus, there are some discrepancies in the evidence regarding the association between APN, AdipoRs, and thyroid cancer, which may be due to the types of thyroid cancer studied, the sample size, research methods, and gender or age composition of the patients.

### The Possible Mechanism by Which APN is Involved in the Pathogenesis of Thyroid Cancer

Thus far, although the exact mechanism of APN underlying thyroid cancer has not been fully elucidated, we have tried to find some clues by reviewing the direct or indirect anti-cancer signaling ways of APN and by combining the existing research data of APN on thyroid cancer. These direct and indirect anticancer pathways of APN are summarized in Figure 3.

### The Direct Anticancer Signaling Pathways of APN/AdipoR

Previous studies have confirmed that APN combined with AdipoR activates or inhibits a series of signaling pathways (summarized in Fig. 3) and promotes tumor protection by promoting apoptosis and cell cycle arrest and inhibiting cell growth, cell proliferation, and cell survival.

AMPK is the most important key molecule in many APN/AdipoR downstream signaling pathways.

AMPK, as a sensor of cellular energy status, is widely found in all eukaryotes, is the master regulator of lipid and glucose metabolism [143] and is activated under low intracellular ATP levels following stresses such as nutritional deficiencies or hypoxia [144, 145]. APN can activate AMPK by upregulating the levels of AMP and liver kinase B1 (LKB1) [146-148]. Importantly, LKB1, also known as serine/threonine kinase 11 (STK11), is an essential upstream activating factor of AMPK. LKB1 phosphorylates and activates AMPK to exert its tumor suppressor function [148, 149]. Moreover, it is worth noting that mammalian target of rapamycin (mTOR) and AMPK have diametrically opposed metabolic effects in eukaryotes. The mTOR is active under nutrient-rich conditions and inactive under nutrient-poor conditions. LKB1-dependent activation of AMPK can directly lead to the phosphorylation of the tumor suppressor tuberous sclerosis complex 2 (TSC2) and, thus, can inhibit mTOR activation, thereby exerting an antitumor effect [147, 150]. In addition, AMPK can also upregulate the expression of p53, p21 and p27 to induce tumor cell growth arrest and apoptosis [151-153]. More importantly, a recent study revealed that the APN-mediated LKB1-AMPK axis participates in the induction of cytotoxic autophagy, leading to tumor inhibition [154].

Similarly, phosphatidylinositol 3-kinase/protein kinase B (PI3K/AKT) is also involved in mediating cellular physiology, including glucose homeostasis, protein synthesis and cell proliferation, as well as cell survival through mediating growth factor signaling pathways. The hyperactivation of this signaling cascade is one of the most common events in human malignant cancers. Inhibition of the PI3K/AKT pathway by APN has been identified. Interestingly, AKT can also inhibit TSC2 and thus neutralize the effects of AMPK [149].

Moreover, c-Jun N-terminal kinase (c-JNK) and extracellular signal-regulated kinases 1 and 2 (ERK1/2) are members of the mitogen-activated protein kinase (MAPK) cascade family, which plays a critical role in stress-induced cell proliferation and apoptosis and performs a function in cell metabolic reprogramming known as the Warburg effect [155, 156]. On the one hand, APN intervention can enhance the c-JNK signaling pathway and cause caspase-3-induced apoptosis. On the other hand, APN intervention can also reduce cell viability and promote cell apoptosis by inhibiting the ERK1/2 signaling pathway.

Furthermore, some studies also suggest that APN plays a role in regulating tumor cell growth and proliferation and the cell cycle through inhibition of signal transducer and activator of transcription 3 (STAT3), wingless-type protein (Wnt), and ubiquitin-specific protease 2 (USP2). Previous studies have demonstrated that constitutively activated STAT3 signaling directly contributes to oncogenesis by stimulating cell proliferation, preventing apoptosis and inhibiting antitumor immunity [157-159]. APN or APN receptor agonist intervention has been shown to inhibit STAT3 signaling to antagonize the role of leptin in tumor cells [160, 161]. In addition, the Wnt/β-catenin signaling pathway has been shown to play an important role in tumor cell proliferation [162]. Wnt signals can inhibit the phosphorylation of β-catenin by glycogen synthase kinase 3 beta (GSK3β), allowing accumulation of unphosphorylated β-catenin in the nucleus and thereby stimulating the expression of target genes such as c-myc, c-jun, and cyclinD1 [162]. APN intervention can inhibit the Wnt/β-catenin signaling pathway by upregulating Wnt inhibitory factor1 (WIF1) or inhibiting GSK3β phosphorylation [163]. The ubiquitin-proteasome system (UPS) is one of the most important protein degradation pathways, is required to maintain the steady state of intracellular proteins and is involved in the regulation of cell apoptosis and the cell cycle [164]. Increasing evidence has indicated that alteration of the UPS plays an important role in cancer development [165, 166]. In particular, USP2 can regulate the expression of cyclin D1, and overexpression of the latter has carcinogenic effects [167, 168]. APN can inhibit the expression of USP2 in tumor cells and promote the degradation of cyclin D1 [169].

In addition, APN can upregulate Bax expression and downregulate Bcl-2 expression to achieve cell cycle arrest and promote apoptosis [170].

Although several studies have been conducted, little progress has been made on APN and its signaling pathway in thyroid cancer to date. Research by Mitsiades et al. [128] showed that APN intervention has no effect on the expression of p53 and p21 in thyroid cancer cell lines (SW579 and BHP7). Cheng et al. [142] found that AdipoR1 is weakly expressed and AdipoR2 is moderately expressed in the papillary thyroid cancer cell lines K1 and B-CPAP and that APN intervention can activate AMPK phosphorylation in cells.

Therefore, more related follow-up studies focusing on APN and its signaling pathways as well as their effects on cell function remain to be conducted.

### Other Possible Indirect Pathways

In addition to the direct effects described above, obesity-related hyperinsulinemia and/or the insulin-like growth factor (IGF) axis might be indirectly involved in the relationship between APN and thyroid cancer.

Increasing evidence from both large cohort studies and animal models has suggested that endogenous hyperinsulinemia is dramatically correlated with the risk of and mortality from several cancers [171]. Although some possible mechanisms explaining this link have been explored, the abnormal IGF system is a specific issue that should be noted [172].

The IGF system includes ligands (including insulin, IGF-1 and IGF-2), receptors (including insulin receptor A/B and IGF receptor 1/2), and IGF-binding protein (IGFBP-1-6), and the importance of this system in regulating cell proliferation, differentiation, and apoptosis has been comprehensively studied [173]. Abnormal IGF signaling has been confirmed to be related to various malignant tumors, such as those related to breast cancer [174], pancreatic cancer [175], and bladder cancer [176], and is usually associated with poor prognosis [177, 178].

It has been increasingly recognized that aberration of the IGF axis plays a complex role in the pathogenesis and progression of thyroid cancer [177, 178]. Although IGF-1 and IGF-1R are both expressed in normal thyroid tissue and thyroid cancer tissue, compared with normal tissue, IGF-1 and IGF-1R are overexpressed in TCs [179]. Moreover, a study conducted by Schmidt et al. [180] showed that serum IGF-1 levels are positively correlated with the risk of DTC.

More importantly, a significant feature of the IGF axis is that it can interact with insulin and insulin receptors (IR-A and IR-B) and even TSH. On the one hand, hyperinsulinemia may enhance the bioavailability of IGF-1 and IGF-2 by inhibiting the synthesis of IGFBP-1/2 and promoting the production of IGF-1 in the liver [181]. On the other hand, the coexistence of IR-A, IR-B and IGF1R generates hybrid receptors, such as IGF-1R/IR-A or IGF-1R/IR-B. These hybrid receptors bind IGF-1/2 and insulin, thereby promoting cell proliferation and cell adhesion [182]. Data from a previous study showed that thyroid tumor cells, especially when poorly differentiated, can autocrine-IGF-2 and then activate the overexpression of the IR-A isoform, thereby promoting the proliferation and inhibiting the apoptosis of these cells [183].

TSH has a tumor-promoting effect. However, the tumorigenicity of the IGF axis has been confirmed to be partially due to crosstalk with TSH through the ERK and PI3K/AKT pathways [184]. Interestingly, as mentioned above, both the PI3K/AKT and ERK pathways are downstream signaling pathways of APN.

Since APN can increase insulin sensitivity in the body, it is reasonable to believe that the complex synergistic interaction between APN and the IGF system or other various obesity-related biomarkers can induce tumorigenesis and development. However, the specific mechanism needs to be further explored. For example, studies by Warakomski et al. [130] showed that serum APN levels in patients with PTC with metabolic syndrome are significantly lower than those in patients without metabolic syndrome.

Furthermore, considering the mitogenic nature of IGF-1, its interaction with receptors can be regulated by IGFBP-3, and upregulation of IGF-1/IGFBP-3 or a lower level of IGFBP-3 may increase the risk of cancer [172, 185]. Another study conducted by Pazaitou-Panayiotou et al. [134] further explored the connection between thyroid cancer and insulin resistance-related biomarkers, including APN, IGF-1, and IGFBP-3. The results showed that the levels of APN, IGF-1, and IGFBP-3 are not significantly different between PTC, FTC, and MTC but that the ratio of IGF-1/APN and IGF-1/(APN×IGFBP-3) ratio is independently related to tumor size.

## Conclusions

In general, thyroid cancer is becoming the most common endocrine cancer in the world, and exploring its potential risk factors, revealing its specific pathogenic mechanisms and identifying effective interventions are the keys to its prevention and treatment. By reviewing previous studies, we found that obesity undoubtedly increases the risk of thyroid cancer. In addition, the increasing importance of APN, as the adipokine that is most closely related to obesity, has been attached to thyroid cancer. However, the existing data cannot strongly conclude whether APN plays a functional role in thyroid cancer in addition to or independently of excess weight. On the one hand, although most clinical studies have supported that circulating APN is negatively associated with thyroid cancer risk, there is insufficient evidence of APN among different BMI stratifications in thyroid cancer patients. Therefore, further clinical data about APN levels between obese and nonobese thyroid cancer patients, even regarding the progress and outcome of these patients, are useful and necessary. On the other hand, the cross-linking of APN and obesity-related hyperinsulinemia and/or the IGF axis further increases the complexity of the relationship between APN and thyroid cancer, posing a challenge but also providing space for further research.
